# Depressive Spectrum Disorders in Cancer: Diagnostic Issues and Intervention. A Critical Review

**DOI:** 10.1007/s11920-017-0785-7

**Published:** 2017-05-09

**Authors:** Rosangela Caruso, Maria GiuliaNanni, Michelle B. Riba, Silvana Sabato, Luigi Grassi

**Affiliations:** 1 Institute of Psychiatry, Department of Biomedical and Specialty Surgical Sciences, University of Ferrara, Via Fossato di Mortara 64a, 44121 Ferrara, Italy; 2 University Hospital Psychiatry Unit, Integrated Department of Mental Health and Addictive Disorders, S. Anna University Hospital and Health Authorities, Ferrara, Italy; 3 Department of Psychiatry, University of Michigan, Ann Arbor, MI USA; 4 University of Michigan Comprehensive Cancer Center, Ann Arbor, MI USA; 5 Psycho-oncology Program, University of Michigan Comprehensive Cancer Center, Ann Arbor, MI USA

**Keywords:** Psychiatry, Depression, Cancer, Psychopharmacology, Antidepressants, Psychotherapy

## Abstract

Depressive spectrum disorders, including major depression, persistent depression, minor and sub-syndromal depression, and other forms of depressive conditions, such as demoralization, are among the most common psychiatric consequences of cancer patients, affecting up to 60% of patients. In spite of the negative effects and the burden for cancer patients and their families, these disorders often remain under-recognized and undertreated. The present review aims at summarizing the relevant data concerning the diagnostic challenges within the depressive spectrum disorders among cancer patients. Also, the most relevant data relative to integrated intervention, including psychopharmacological and psychosocial treatment, for depression in cancer patients are critically evaluated. It is mandatory that health care professionals working in oncology (e.g., oncologists, surgeons, radiation oncologists, primary care physicians, nurses, social workers, psychologists) receive training in the diagnosis and integrated management of the different types of disorder within the spectrum of clinical depression.

## Introduction

Depression is one of the most common psychiatric disorders in cancer patients [1, 2•]. An extremely vast literature has examined several issues related to depression including its prevalence, with data showing that 25–30% of cancer patients suffer from depressive disorders (percentages ranging from 4 to 60%); [3] the most important risk factors, including biological, psychological, and social factors; and the most effective and valid methods to correctly screen and assess depression in cancer, including short (and ultra-short) tools, self-report questionnaires, and semi-structured interviews [4••]. Depression has been shown to have negative consequences on both the patient and family with data indicating a role of this clinical condition in reducing adherence to treatments, in increasing subjective perception of physical symptoms and, possibly, in worsening prognosis [5, 6•].

Yet depression in cancer patients still remains quite often undetected in clinical practice with studies indicating that in general 50–60% of depressed cancer patients are not properly recognized [7]. Among elderly people, in whom the incidence of both depression and cancer is higher, this problem is even more evident [8]. As reported by Greenberg [9], cultural, stigma, and organizational issues continue to represent an obstacle in the delivery of mental health interventions in cancer settings. It has been reported that communication is also a problem, with a very low proportion of patients discussing their low mood with their doctors or oncologists and choosing to address somatic rather than affective and emotional symptoms [9]. Diagnostic issues are an important cause of under-recognition. In fact, the term “depression” indicates a large-spectrum of conditions, ranging from a transient normal feeling in response to an adverse event, to a highly severe and impairing syndrome. Thus, the concept of depressive spectrum disorders seems to be more appropriate to address the different manifestations of clinical depression, including major depression, persistent depression, minor and sub-syndromal depression, and other forms of depressive conditions, such as demoralization [4••]. Thinking in terms of a spectrum may also help clinicians to be more sensitive to the different forms of depressive conditions in the different phases of the cancer trajectory, and to be more precise in planning proper intervention.

With this background, the aims of the present critical review are (i) to summarize the diagnostic challenges within the depressive spectrum disorders among cancer patients and (ii) to report the most relevant data relative to integrated intervention, including psychosocial and psychopharmacological treatment, for depression in cancer patients.

For these purposes, a search was made of the major databases over the last 15 years (Embase/Medline, PsycLIT, PsycINFO, the Cochrane Library) from January 2001 to June 2016 by including the most relevant studies in full journal articles that investigated the problem of diagnosis and treatment of depression in patients with cancer. Studies published in conference proceedings, qualitative research, commentaries and discussions, letters, books, book chapters, or research not published in the English language were excluded.

## Diagnostic Issues

The appropriate criteria for recognizing and diagnosing depression in cancer patients have been extensively debated over the last years [10, 11]. In cancer settings, it is important to differentiate the types and forms of depression, according to the most commonly used systems in nosology, namely the International Classification of Diseases (ICD) and the Diagnostic and Statistical Manual of Mental Disorders (DSM).

### Major Depression

Major depression is arguably the most severe form of depressive syndromes, affecting about 7% of the general population (8–9% of female and 3–4% of men), with data in oncology indicating a prevalence between 15 and 40%, depending on several factors (e.g., type, stage of cancer, in/outpatient, on/off treatment, type of diagnostic tool for depression) [2•]. A significant problem underlined by a number of authors over the last 35 years is that many symptoms of depression, such as loss of appetite, loss of weight, insomnia, cognitive impairment, fatigue, and loss of energy, may be a consequence of cancer or cancer treatment rather than depression itself. Therefore the debate regarding the need to modify the DSM criteria of major depression when applied to cancer (e.g., [12, 13]) has grown with different opinions and several proposals suggested, namely: to include all the symptoms irrespective of the fact that these symptoms may or may not be attributable to cancer (*inclusive approach*), to replace somatic symptoms with cognitive-affective items (*substitute approach*), to add some new affective symptoms to the original criteria (*alternative approach*), and to exclude somatic symptoms and use only affective symptoms to make the diagnosis (*exclusive approach*) [14•] (Table 1). In a study of 728 cancer patient with major depression, Akechi et al. [15] showed that the somatic symptoms (appetite loss, weight loss, insomnia, fatigue, loss of energy, and diminished ability to think or concentrate), feelings of worthlessness, and suicidal ideation were not useful, among the DSM-IV criteria, for diagnosing and/or judging the severity of depression among cancer patients. Two other alternative criteria (“not participating in medical care in spite of ability to do so, not progressing despite improving medical condition and/or in functioning at a lower level than the medical condition warrants”; “social withdrawal or decreased talkativeness”) showed to be useful markers of the severity of major depression among cancer patients, suggesting the need for a two-step diagnostic approach (inclusive criteria plus two alternative criteria). More recent data, however, indicate that many somatic symptoms remain influential when diagnosing major depression in the context of cancer, with scoring-method not excluding somatic symptoms having the greatest effect on assessment outcomes [16]. In palliative care, in particular, the need to have a clear-cut framework to assess depression has frequently been balanced with the need to focus on clinical utility and evaluation of symptoms and depressive syndromes rather than on a specific diagnosis, such as major depression [17, 18]. However, in a recent study involving 969 patients with advanced cancer within the European Palliative Care Research Collaborative-Computer Symptom Assessment Study (EPCRC-CSA) [19••], it has been shown that the prevalence of depression changed according to the scoring-methods used, specifically inclusive (algorithm scoring including the somatic symptom-criteria, rate 13.7%), exclusive (algorithm scoring excluding the somatic symptom-criteria, rate 14.9%), and sum-score (sum of all symptoms of the Patient Health Questionnaire cut-off ≥8, rate 45.3%). The scoring-method, not excluding somatic symptoms, had the greatest effect on assessment outcomes, since depression was significantly associated with pain and lower performance status. Also, regarding the survivorship phase, in a large study of 3,550 cancer survivors (aged >18 years) and 26,917 adults without cancer (2010 Behavioral Risk Factor Surveillance System, Depressive symptoms), 13.7% of cancer survivors (vs. 8.9% of adults without cancer) reported having major depression [20••].

**Table 1:** Proposals for the diagnosis of major depression in cancer patients [12, 13, 15]

Etiologic approach (ICD-DSM): based on the nine traditional symptoms of major depression (depressed mood, anhedonia, appetite or weight change, sleep disturbance, fatigue, psychomotor disturbance, feelings of worthlessness or guilt, impaired concentration, and suicidal thoughts) to be present (at least five—of which either depressed mood or anhedonia are mandatory—for at least 2 weeks of duration), but symptoms that are clearly and fully attributable to the cancer (as a general medical condition) are excluded, otherwise depression due to a general medical condition is diagnosed.
Inclusive approach (modified ICD-DSM): as above, but symptoms are counted regardless whether or not they might be attributable to cancer.
Substitutive approach (Endicott’s criteria) [12]: in Criterion A, neurovegetative/somatic symptoms are substituted by cognitive/affective symptoms (specifically: *diminished ability to think or concentrate or indecisiveness* replaced by *fearfulness or depressed appearance in face or body posture*; *weight loss or gain or a decrease in appetite* replaced by *social withdrawal or decreased talkativeness*; *insomnia or hypersomnia* replaced by *brooding*, *self-pity or pessimism*; *fatigue or loss of energy* replaced by “*cannot be cheered up, doesn’t smile, no response to good news or funny situations*). These substitute symptoms should be used if the medical condition is likely to affect/cause the somatic symptoms.
Exclusive approach (Cavanaugh’s criteria) [13]: in Criterion A, *hopelessness*, *helplessness*, and *not caring anymore* are added to either depressed mood or anhedonia as mandatory (plus three other symptoms) for at least 2 weeks of duration; significant weight loss or weight gain, psychomotor agitation or retardation, insomnia or hypersomnia, and fatigue or loss of energy are excluded, unless temporarily related to cognitive/affective symptoms. In Criterion B, one extra item (*not participating in medical care, in spite of ability to do so, he is not progressing despite improved medical condition and/or is functioning at a lower level than the medical condition warrant)* is added.
Alternative approach (Akechi’s criteria) [15]: as in inclusive approach plus other items do be fulfilled in order to diagnose mild major depressive episode (*fearfulness or depressed appearance in face or body posture*; *brooding*, *self-pity or pessimism*), moderate major depressive episode (*not participating in medical care in spite of ability to do so*, *not progressing despite improving medical condition and/or in functioning at a lower level than the medical condition warrants*; *social withdrawal or decreased talkativeness*) and severe major depressive episode (*cannot be cheered up, doesn’t smile, no response to good news or funny situations).*

### Persistent Depressive Disorder

Protracted forms of depression have been usually conceptualized in psychiatric nosology (ICD-10, DSM-IV) as persistent depressive conditions (e.g., dysthymia; chronic specifier of major depressive episodes), which have been merged into Persistent Depressive Disorder (PDD) by including both chronic major depressive disorder and the previous dysthymic disorder in DSM-5. However, as recently indicated [21], research findings suggest that dysthymic disorder is a heterogeneous diagnosis encompassing many different depressive (and anxiety or personality weighted) conditions that can determine a heterogeneous domain diagnosis of PDD with possible risks in terms of the validity as of this diagnostic entity. Data in cancer settings are scarce, besides the few studies carried out through psychiatric interview showing a prevalence of about 2–3% of dysthymia in cancer patients, [22] that became 15% when major depression was considered (double depression: major depression plus dysthymia) [23••]. The diagnostic criteria are in any case not easy to follow in cancer patients and, again, some proposals have been advanced to change or modify those criteria [13], but without any data indicating if that has been clinically useful (see Table 2 for details).

**Table 2:** Proposals for the diagnosis of dysthymia and adjustment disorders in cancer patients [13]

Dysthymia
Based on the presence of depressed mood for the last 2 years (criterion A) plus at least two of the six traditional symptoms of dysthymia (Criterion B), defined in DSM5 as Persistent Depressive Disorder (i.e., poor appetite or overeating; insomnia or hypersomnia, low energy or fatigue, low self-esteem, poor concentration or difficulty making decisions, feelings of hopelessness), but after having ascertained that (a) hopelessness is not demoralization or discouragement related to the reality of the medical illness; (b) low self-esteem is feeling bad about oneself, not the situation; and (c) all the somatic symptoms are not easily explained by physical illness, treatments, or hospital environment.
Adjustment disorders (with depressed mood)
In Criterion A, The development of depressed mood in response to an identifiable stressor(s) occurring within 3 months of the onset of the stressor(s) should include illness (e.g., cancer) or treatment, as stressor(s).
In Criterion B, the fact that there is a marked distress reaction in excess of what would be expected from exposure to the stressor should consider that rarely with medical illness is the distress considered in excess of the stressor. Also, a further item (*treatment is recommended)* is added.

### Adjustment Disorder and Other Depressive Disorder

In spite of the high prevalence of adjustment disorders in cancer settings (20-25%) [3, 24], the diagnostic features and the clinical utility of diagnosing these disorders with both depressed mood or mixed features (anxiety and depression) in cancer, as well as in medically ill patients in general, have been repeatedly questioned. The main characteristics of adjustment disorder and its sub-types have been reported to be vague and imprecise to have any clinical utility, particularly in oncology [25–27]. The lack of guidance on the distinction from normal to abnormal stress reactions; plus the lack of specific diagnostic criteria in terms of symptom numbers, behavioral parameters, or their combinations; and the inadequate operationalization to distinguish adjustment from adaptation, socialization, and coping have been reported [28, 29]. Although the criteria were proposed to be slightly modified in cancer settings [13] (Table 2) and although in the DSM-5 the disorder is now classified under the chapter of Trauma and Stress-Related Disorders, its definition and diagnostic criteria are still criticized [30]. By examining patients with the diagnosis of adjustment disorders and major depression, it has been reported that many different sub-types (e.g., adjustment disorders with features of alexithymia, major depression with irritable mood) are possible in medically ill, including cancer patients, but this does not mean that all these phenomenological conditions are separate and represent specific diagnoses [31, 32].

For the same reasons, the DSM-5 and ICD-10 categorical distinction between minor depression, mixed anxiety-depressive disorders, and brief recurrent depressive episodes, based on quite debatable criteria, are also not particularly useful in cancer and palliative care settings, where the duration of suffering, the impact of the stress represented by cancer and treatment, and the intensity of depressive symptoms may fluctuate and cannot be represented by restrictive categorical criteria such as number of symptoms, and days of duration) of DSM5.

### Demoralization

Demoralization, which is also part of depressive spectrum disorders but not detectable by using ICD and DSM psychiatric nosology, [33] has been carefully examined and described over the last 20 years (Table 3). de Figueiredo [34, 38] clearly underlines the differences between demoralization and major depression, indicating that while patients with major depression perceive the source of distress within themselves, have feelings of guilt and anhedonia, and do not perceive any motivation, those with demoralization perceive the source of distress outside the self, do not feel guilty (but subjectively incompetent to cope), do not present anhedonia, and feel uncertain about the direction one’s actions should take, but the magnitude of motivation is intact. Fava et al. [35], within the Diagnostic Criteria for Psychosomatic Research (DCPR), also describe demoralization as a condition (or dimension) characterized by a sense of failure to have met one’s own expectations, being unable to cope with some pressing problems, and feelings of helplessness/hopelessness or giving up. The demoralization syndrome has been proposed as a specific clinical entity if a series of criteria are met [36, 37], and more recently the DCPR criteria for demoralization has been revised to be more useful both for research and clinical care [38••] (see Table 3 for details). Also, research criteria have been proposed to facilitate the assessment of this construct in the medically ill [33]. The prevalence of demoralization, as a clinical syndrome separated from major depression, among 807 Italian medically ill patients recruited from different settings including oncology, was demonstrated to be 30.4%, while major depression (according to the DSM-IV) was present in 16.7% of the patients [39, 40]. Similar data were reported in a study of 721 patients with severe medical illness, including advanced cancer [41]. Demoralization has been shown to have a negative impact on cancer patients, such as poorer quality of life and higher levels of worries and preoccupation related to cancer (e.g., the illness itself, feeling different from others, the impact on sexual life), [42] loss of dignity, [43], and suicidal ideation [44]. Several recent systematic reviews on this topic are available [45•, 46•, 47] and confirm the importance of this syndrome in medical and oncology settings [48].

**Table 3:** Proposed criteria for demoralization in the medically ill, including cancer patients

Demoralization/subjective incompetence (De Figueiredo) [34]
A combination of distress (anxiety, sadness, discouragement, and resentment) and subjective incompetence (a feeling of being trapped or blocked because of a sense of inability to plan and initiate concerted action toward one or more goals)
Persistent failure of coping with internally or externally induced stress
Feelings of impotence, isolation, and despair
Individual’s self-esteem damaged
Feelings of rejection by others because of his or her failure to meet their expectations
Diagnostic Criteria for Psychosomatic Research (DCPR Criteria)—demoralization module (Fava et al.) [35]
A. A feeling state characterized by the patient’s consciousness of having failed to meet his or her own expectations (or those of others) or being unable to cope with some pressing problems; the patient experiences feelings of helplessness, or hopelessness, or giving up
B. The feeling state should be prolonged and generalized (at least 1 month duration) C. The feeling closely antedated the manifestations of a medical disorder or exacerbated its symptoms
DCPR Criteria - demoralization module -Revised (Fava et al.) [38••]
A. A feeling state characterized by the perception of being unable to cope with some pressing problems and/or of lack of adequate support from others (helplessness); the individual maintains the capacity to react
B. The feeling state is prolonged and generalized (duration of at least 1 month) C. A feeling state characterized by the consciousness of having failed to meet expectations associated with the conviction that there are no solutions for current problems and difficulties (hopelessness)
[criteria A and B are required; criterion C is a specifier for the presence of hopelessness]
Demoralization syndrome (Kissane et al.) [36, 37]
Encompassing hopelessness or loss of meaning and purpose in life
Cognitive attitudes of pessimism, helplessness, sense of being trapped, personal failure
Absence of drive or motivation to cope differently
Associated features of social alienation or isolation and lack of support
Fluctuation in emotional intensity
Persistence of above-mentioned phenomena across two or more weeks (and a major depressive or other psychiatric episode should not be present as the primary condition)

## Intervention

Intervention for depressive spectrum disorders has been examined in a number of studies, with several reviews and meta-analyses available. Some of them concentrate attention only on the proper use of psychotropic drugs, including antidepressants (ADs) and/or adjuvant drugs [49•, 50••, 51•], some only on psychological and psychotherapy interventions [52••], and some on both [53–56]. It is actually the integration of these two forms of intervention, as the definition of “PsychopharmOncology” [47] indicates, that is important to be considered in the care of cancer patients.

### Psychopharmacological Intervention

According to psychopharmacological studies, there is evidence that ADs are more effective than placebo in both cancer patients with major depression or depressive symptoms and in those with other cancer-related distressing symptoms (see [49•,^50^••,^51^•] for specific reviews and [57, 58]). Their efficacy is positively associated with length of treatment, and no difference has been reported between ADs and placebo in terms of overall acceptability [59•, 60]. The data regarding the use of ADs in cancer patients is however quite poor for several reasons, such as lack of RCTs, high number of uncontrolled clinical trials, lack of longer follow-up period (more than 12 weeks), imprecision arising from small sample sizes and wide confidence intervals, and inconsistency due to statistical or clinical heterogeneity [52••].

It is also a fact that a high number of cancer patients with depression did not receive proper antidepressant treatment. A recent review of prescriptions of ADs in oncology indicate that there is considerable variation in the prescribing patterns across the world, with only few studies reporting robust data on exact dose or follow-up regimens [61••]. This confirms recent reports by Walker et al. [62••] showing that 1,130 (73%) out of 1,538 patients with depression were not receiving effective treatment, and by Fisch et al. [63•] showing that among 3,106 ambulatory patients with cancer, only one fourth of those with depression were treated with ADs. Also, a further German study showed that a lower proportion of cancer patients with depression had received ADs (66.5%) in comparison with a control group of patients with only depression (72.8%) [64]. There are also variables influencing the prescription of ADs, including gender (females > men), ethnicity (Caucasians receiving more SSRIs than other ethnic groups), and treatment (> in patients with extensive cancer treatment) [56, 65, 66]. In contrast with these data, however, a different study carried out in Australia indicated that newest ADs were more commonly prescribed in cancer populations, particularly if in an advanced phase of illness, than in cancer-free populations [67].

### Psychosocial Intervention

With regard to psychosocial intervention, an extremely high number of studies, meta-analyses, and reviews exist. The types of psychotherapy approaches in cancer patients with depression are vast (from cognitive-behavior therapy to existential-based intervention, from psychodynamically oriented therapy to educational, relaxation, expressive-supportive therapy, and other forms of treatment, such as mindfulness), supporting the view that it is more important to focus on participants’ variables (e.g., level of depressive symptoms, type and stage of cancer, personality variables) [68] rather than the type of intervention itself [69, 70]. For example, a systematic review [71] examining 199 potential papers, of which a total of 20 studies were selected and involved 3,340 heterogeneous cancer patients, showed that cancer patients who had the greater benefit from intervention were those who reported low levels of optimism and neuroticism; high levels of emotional expressiveness, interpersonal sensitivity, and dispositional hypnotisability; and poorer quality of life, interpersonal relationships, and sense of control.

The effectiveness of psychosocial interventions for cancer patients was shown to be related to many different variables, including the type of participants (e.g., cancer type and stage, inpatients or outpatients), the mode of delivery of the intervention (e.g., face-to-face, telephone psychological support), and the discipline of the therapist (e.g., nurses with training in psycho-education, psychologists) [72]. However, among advanced cancer patients with depression, a Cochrane review by Akechi et al. [73] indicated that there is evidence from randomized controlled trials (RCTs) suggesting that psychotherapy is useful for treating depressive states, although there is a lack of specific studies examining the effectiveness of psychotherapy for patients with clinically diagnosed depression.

This many-sided scenario has determined some inconsistencies in the reported effects of psychosocial intervention, with authors emphasizing its significant benefits [74–77], and others suggesting to be more cautious in examining the results of the studies available [78, 79]. Also, disagreement exists about the format of psychotherapy, with some authors underlining the superiority of individual treatments versus group therapy [80], and others suggesting the equivalence in efficacy of individual and group psychotherapy [81]. In a systematic review of 14 published meta-analyses, Linden and Girgis [82] underscored that treatment effects are consistently positive but also vary greatly in magnitude, even if there is no evidence for differential treatment effects for different cancer types and stages. More recently, in a vast review analyzing 98 studies (covering 22,238 patients) with 218 treatment-control comparisons, Faller et al. [48] observed a significant small-to-medium effect for individual and group psychotherapy and psycho-education, with effects sustained in the medium term (≤6 months) and long term (>6 months).

Less data are available regarding the efficacy of psychosocial intervention in demoralization, although some recent RCTs in patients with both advanced and non-stages of illness indicate that a meaning-oriented and dignity-oriented therapy significantly reduce demoralization scores and improve psychosocial functioning [83••, 84, 85].

### Integrated Approaches

The need for the integration of psychosocial and pharmacological intervention within specific programs has also emerged in the last years [86]. A review relative to 10 studies involving 1,362 severely depressed cancer patients with mixed cancer types and stages confirmed that integrated psychological and pharmacologic approaches are effective but should be targeted productively toward cancer patients with elevated depressive symptoms in order to maximize effectiveness, accessibility, and integration into clinical care [87]. Significant benefits in terms of outcome and cost-effectiveness have been shown in a recent series of studies within the Depression Care for People with Cancer (DCPC) program in the Symptom Management Research Trials (SMaRT) Oncology randomized controlled trials carried out in Scotland [88–90]. More specifically, in one study [91••] that enrolled 500 participants of whom 253 were randomly allocated to the DCPC program and 247 to usual care (UC), response to treatment was higher among the former than the latter (62% vs. 17%). Patients in the DCPC program had less depression, anxiety, pain, and fatigue and better functioning, health, quality of life, and perceived quality of depression care than patients in UC. In a further study, the same authors [92] confirmed their results when examining the efficacy of the DCPC program in the management of depression in lung cancer patients compared with UC. A recent report updating the Cancer Care Ontario Program, an Evidence-Based Care guideline [93•] for the management of depression in adult patients with cancer, specifically has focused on integrating practical management tools to assist clinicians in delivering appropriate treatments for depression in patients with cancer by covering pharmacologic, psychological, and collaborative care interventions. More recently, the same group [94•] has confirmed that a collaborative care model that incorporates a stepped care approach is recommended with multidisciplinary mental health care restructuring be required for optimal management of depression.

## Conclusions

Depressive spectrum disorders in cancer patients should be taken into serious consideration since in patients with medical diseases, depression has the largest effect on worsening mean health scores and on increasing disability compared with the other chronic conditions [95]. However, the several clinical and phenomenological aspects of depressive disorders should be re-examined and recognized in terms of “spectrum” in order to screen the patients in a more specific way and to reach a more reliable diagnosis which makes referral and intervention more appropriate.

Recent consensus guidelines, algorithms, and reviews have been developed and proposed in the hope to promote integrated psychopharmacological and psychosocial treatment [96–99] in order to reduce the burden of psychiatric morbidity on both the patient and the family [100].

Thus, it is mandatory that health care professionals working in oncology, such as oncologists, surgeons, radiation oncologists, primary care physicians, nurses, social workers, and psychologists, receive training in the diagnosis and management of depressive spectrum disorders, given the different intervention approaches according to the type of depressive disorder [101, 102].

It has to be stressed that cancer patients with depression have been reported to have a low rate of utilization of mental health services [103–106], although some improvement has been recently underscored in some countries such as the USA [107]. In many other countries, significant disparities and inequalities still exist as far as psychosocial care of cancer patients is concerned [108, 109••].

Therefore, more research and more efforts in the development of psycho-oncology services and in integration of mental health in oncology are necessary in cancer and palliative care settings.
